# Molecular Diversity and Comparison of Staphylococcal Cassette Chromosome *mec* Element in Hospital‐ and Community‐Associated Methicillin‐Resistant *Staphylococcus aureus* From Pakistan

**DOI:** 10.1155/ijm/5513694

**Published:** 2026-06-08

**Authors:** Asad Ali, Saman Jabbar, Shahid Aziz, Saba Riaz

**Affiliations:** ^1^ Institute of Microbiology and Molecular Genetics, University of the Punjab, Lahore, Punjab, Pakistan, pu.edu.pk; ^2^ Institute of Allied Health Sciences, Wah Medical College, National University of Medical Sciences, Rawalpindi, Punjab, Pakistan, numspak.edu.pk; ^3^ CitiLab and Research Centre, 525-A Faisal Town, Lahore, Punjab, Pakistan

**Keywords:** antibiotic resistance, MRSA, SCC*mec*

## Abstract

**Background:**

Methicillin‐resistant *Staphylococcus aureus* (MRSA) is one of the leading causes of hospital and community‐acquired infections, with the increasing threat of antimicrobial resistance worldwide. Staphylococcal Cassette Chromosome *mec* (SCC*mec*) typing is one of the molecular techniques to predict the genetic background of MRSA strains and predict the level of antibiotic resistance. This study aimed to find out and compare the predominant SCC*mec* types in the hospital and community settings along with antibiotic resistance burden.

**Methods:**

A total of 295 MRSA isolates were recovered from the hospital and community settings in Lahore, Pakistan. The growth characteristics of the isolates were initially determined using Mannitol salt agar (MSA). Isolates were then subjected to further biochemical confirmation by catalase, DNase and tube coagulase tests according to standard operating procedure used in Pakistan. MRSA screening was performed using Cefoxitin 30 *μ*g disc. Antibiotic resistance patterns were determined against commonly used antibiotics, and the results were interpreted according to Clinical and Laboratory Standards Institute (CLSI) guidelines. Molecular detection of MRSA was done by *mecA* gene, and a multiplex PCR was used to determine the prevalent SCC*mec* types (I‐VI).

**Results:**

A total of 145 (49.15%) MRSA isolates were identified. SCC*mec* type III was predominant (39.31%, *n* = 57), while no type V was detected. Antibiotic resistance profiles revealed the highest resistance towards ciprofloxacin (78.62%), while high susceptibility rates were recorded for ceftaroline (95.17%), quinupristin/dalfopristin (93.79%), and linezolid (91.03%). Ciprofloxacin resistance was significantly associated with SCC*mec* type III. Type I SCC*mec* was associated considerably with samples originating from clinical sources.

**Conclusions:**

Prevalence of MRSA and the types of SCC*mec* are on the rise. Because of the high prevalence of multiresistant type III cassettes, antibiotic resistance is also increasing. The high occurrence of community‐specific SCC*mec* in hospital settings suggests implementing infection control practices to prevent severe MRSA infection and the rise in antibiotic resistance.

## 1. Introduction

*Staphylococcus aureus* is a highly opportunistic pathogen, usually as a normal flora on half of the adult skin and about 15% persistently carrying in their anterior nares [1]. It causes several human diseases ranging from toxin‐mediated infections to pyogenic and invasive infections [2, 3]. Besides causing nosocomial infections, MRSA is now frequently seen in community‐associated (CA) infections [4]. CA‐MRSA possesses Panton–Valentine Leukocidin (PVL) toxins that are pore‐forming proteins, allowing *S. aureus* to damage and invade host cells to establish infection and induce the release of chemotactic factors. PVL production is related to fasciitis, necrotizing pneumonia, skin and soft tissue infections and necrosis and apoptosis of leukocytes [5]. Successful treatment of such infections began with the commercialization of penicillin in the 1940s, which acted as the drug of choice until the emergence of resistant strains through the acquisition of beta‐lactamase plasmid. Treatment of penicillin‐resistant *S. aureus* infections included semisynthetic penicillin drugs, such as methicillin. However, in the 1960s, the rise of methicillin‐resistant *S. aureus* (MRSA) strains was apparent [3, 6].

Typically, *S. aureus* encodes a methicillin‐susceptible penicillin‐binding protein (PBP) as a cell wall component. The *mecA* encodes altered penicillin‐binding protein 2a (PBP‐2a) positioned in the bacterial cell wall, with low affinity for *β*‐lactams [7]. This resistance to beta‐lactams is encoded on a mobile genetic element (MGE) known as staphylococcal cassette chromosome *mec* (SCC*mec*), which also encodes resistance to several other classes of antibiotics, including fluoroquinolones, tetracyclines, aminoglycosides etc. [4, 8, 9]. The genetic architecture of SCC*mec* reveals three distinct parts: *mec* gene complex, *ccr* gene complex, and junkyard or joining (J) region. The *mec* complex harbors *mecA* responsible for methicillin‐resistant phenotype while *ccr* complex encodes cassette chromosome recombinases (*ccr*) that help in the excision and integration of SCC*mec* into *S. aureus* chromosome. The *mec* and *ccr* complexes are flanked on either side by J‐regions (J1, J2, and J3), constituting a nonessential part of *mec* element but also containing antibiotic resistance genes. Different classes of *mec* gene complex have been reported and named A to E. Similarly, several *ccr* gene complex allotypes have been reported and named 1 to 8. A combination of different *mec* complex classes and *ccr* gene allotypes has defined various SCC*mec* types. Thus, 15 different SCC*mec* types (I–XV) have been assigned for *S. aureus* till now, which are further divided into subtypes based on differences in J‐regions [10–12].

SCC*mec* typing is a reliable tool for identifying genetic relatedness and epidemiology of MRSA clones circulating worldwide, which is particularly important in emerging MRSA clones that are frequently evolving and spreading around the globe. MRSA is distributed worldwide, and its prevalence is reportedly 13.3%–36.1% in Pakistan, varying across different study periods [13–15]. The most prevalent SCC*mec* types being reported from clinical settings include Types I, II, III, and IV [13, 16–19]. However, SCC*mec* prevalence data is scarce, and updated epidemiology is required from diverse locations for proper infection control policy development. The current study was designed to find SCC*mec* types of MRSA prevailing in the hospital and community settings and the updated antibiotic resistance profiles associated with the prevalent SCC*mec* types in Lahore, Pakistan.

## 2. Materials and Methods

### 2.1. Bacterial Isolates

This cross‐sectional study included 295 clinical and nonclinical samples collected randomly during August 2021 and July 2022. The clinical MRSA samples (*n* = 119) were collected from a tertiary care hospital in Lahore, Pakistan, and the nonclinical *S. aureus* samples (*n* = 176) came from the community origin, including hands and nares swabs from the food handlers, community and environment as per the established case definition of CA‐MRSA [20]. The study’s 1‐year duration provided sufficient time for a good representation of the collected isolates.

### 2.2. Characterization of Isolates and Antibiotic Sensitivity Profile

All the collected samples were first screened for characterization as *S. aureus* through various biochemical tests, including characteristic growth on mannitol salt and blood agar, catalase, coagulase, and deoxyribonuclease (DNase) activities. The control strains used for biochemical identification included *S. aureus* as positive, while *Enterococcus faecalis*, *S. epidermidis*, and *E. coli* for catalase, coagulase, and DNase tests, respectively. Phenotypic detection of MRSA was done by screening the isolates for resistance towards the cefoxitin antibiotic disc (FOX‐30 *μ*g). Besides, antibiotic resistance profiles for all the isolates were also established against 13 different antibiotics discs (Oxoid, England) including gentamicin (CN‐10 *μ*g), ciprofloxacin (CIP‐5 *μ*g), trimethoprim‐sulphamethoxazole (SXT‐25 *μ*g), clarithromycin (CLR‐15 *μ*g), ceftaroline (CPT‐30 *μ*g), fusidic acid (FA‐10 *μ*g), tigecycline (TGC‐15 *μ*g), clindamycin (DA‐2 *μ*g), erythromycin (E‐15 *μ*g), linezolid (LZD‐30 *μ*g), chloramphenicol (C‐30 *μ*g), quinupristin/dalfopristin (QD‐15 *μ*g), and tetracycline (TE‐30 *μ*g). Antibiotic susceptibility testing (AST) was done on Mueller–Hinton agar (MHA) media (Oxoid, England) according to Kirby–Bauer disc diffusion method, following Clinical and Laboratory Standards Institute (CLSI) 2019 guidelines, and interpreting the sensitivity profile [21], except for fusidic acid and tigecyclines that were interpreted per European Committee on Antimicrobial Susceptibility Testing (EUCAST) guidelines v10.0 [22].

### 2.3. Molecular Detection of MRSA and SCCmec Typing

The *mecA* gene was detected using previously described primers and PCR conditions (Table 1). A multiplex PCR was conducted to detect the major SCC*mec* types I–VI using previously designed primers (Table 1) with specified PCR conditions. The PCR reaction was carried out using 12.5 *μ*L of PCR Buffer pre‐mix (2× Fermentas Master Mix, Thermo Scientific, United States), 5 *μ*L of DNA template obtained by boiling, 0.5 *μ*L of each 20 *μ*M primer and nuclease‐free water up to the final reaction volume of 25 *μ*L. The PCR conditions included initial denaturation at 95°C for 10 min, 35 cycles of denaturation for 30 s at 95°C, annealing for 30 s at 55.51°C, extension for 1 min at 72°C, and final extension at 72°C for 10 min. The reaction was carried out in a T100 thermocycler (Bio‐Rad, USA).

**Table 1:** Oligonucleotide primers used in the current study.

S. no.	Primers	Oligonucleotide sequence (5 ^′^‐3 ^′^)	Specificity	Amplicon size	Reference
1.	*mecA*	F: TGCTATCCACCCTCAAACAGG	*mecA*	285 bp	[23]
		R: AACGTTGTAACCACCCCAAGA			
2.	*CIF2*	F: TTCGAGTTGCTGATGAAGAAGG	SCC*mec* Type I	495 bp	[24]
		R: ATTTACCACAAGGACTACCAGC			
3.	*KDP*	F: AATCATCTGCCATTGGTGATGC	SCC*mec* Type II	284 bp	
		R: CGAATGAAGTGAAAGAAAGTGG			
4.	*MECI*	F: ATCAAGACTTGCATTCAGGC	SCC*mec* Type II, III	209 bp	
		R: GCGGTTTCAATTCACTTGTC			
5.	*DCS*	F: CATCCTATGATAGCTTGGTC	SCC*mec* Type I, II, IV, VI	342 bp	
		R: CTAAATCATAGCCATGACCG			
6.	*RIF4*	F: GTGATTGTTCGAGATATGTGG	SCC*mec* Type III	243 bp	
		R: CGCTTTATCTGTATCTATCGC			
7.	*RIF5*	F: TTCTTAAGTACACGCTGAATCG		414 bp	
		R: GTCACAGTAATTCCATCAATGC			
8.	*SCCmec V J1*	F: TTCTCCATTCTTGTTCATCC	SCC*mec* Type V	377 bp	[25]
		R: AGAGACTACTGACTTAAGTGG			
9.	*ccrB2*	F: AGTTTCTCAGAATTCGAACG	SCC*mec* Type II, IV	311 bp	
		R: AGTTTCTCAGAATTCGAACG			

### 2.4. Statistical Analysis

For categorical variables, the association between different variables was accessed using chi‐square analysis. A binary logistic regression model was used to conducted the association between various categorical variables with binary parameters for evaluating independent effect of SCC*mec* types on antibiotic resistance. A *p* value of <0.05 was considered statistically significant.

## 3. Results

### 3.1. Demographics and Prevalence of the Collected MRSA Samples

Of the 295 S*. aureus* samples under investigation, 119 were exclusively of clinical origin. Out of 176 nonclinical samples, 50 *S. aureus* were recovered in which only 26 (14.77%) were MRSA and were included in further analysis, accumulating to 145 MRSA isolates. For the CA‐MRSA, 16 (61.54%) isolates were from males and 10 (38.46%) were from female. For the sample source, 15 (69.23%) isolates were detected from the hands/skin and eight (30.77%) were from the nares. All the isolates came from individuals aged 1–80 years. It was observed that the highest rate (46.9%, *n* = 68) of MRSA colonization of infection was in the age group 21–40 years, while the lowest rate (7.59%, *n* = 11) was in the age group 61–80 years. MRSA was higher in males (64.14%, *n* = 93) than in females (35.86%, *n* = 52). The bacterial isolates came from different clinical samples, including blood, pus, urine, sputum, cerebrospinal fluid (CSF), tracheal secretion, etc. MRSA was highly prevalent in pus (62.76%, *n* = 91), followed by blood (5.52%, n = 8). The nonclinical MRSA isolates detected the highest occurrence on hands and nares (each 4.83%, *n* = 7). Only three isolates were recovered from the environment (Table 2).

**Table 2:** Description of MRSA isolates used in this study.

Collected sample type (*n*)	Recovered specimen (*n*)	MRSA [*n* (%)]
Normal population (100)	Nasal (23 both)	7 (4.83)
	Skin	7 (4.83)
Food handlers (80)	Nasal (19 both)	2 (1.38)
	Hands	7 (4.83)
Environment (20)	Environmental (8)	3 (2.07)
Clinical samples (119)	Pus	91 (62.76)
	Blood	8 (5.52)
	Tissue	4 (2.76)
	CSF	3 (2.07)
	Sputum	3 (2.07)
	Fluid	2 (1.38)
	Foley tip	2 (1.38)
	Tracheal secretion	2 (1.38)
	Urine	1 (0.69)
	Ear swab	1 (0.69)
	HVS	1 (0.69)
	Knee joint	1 (0.69)

### 3.2. Antibiogram

**Figure 1:**
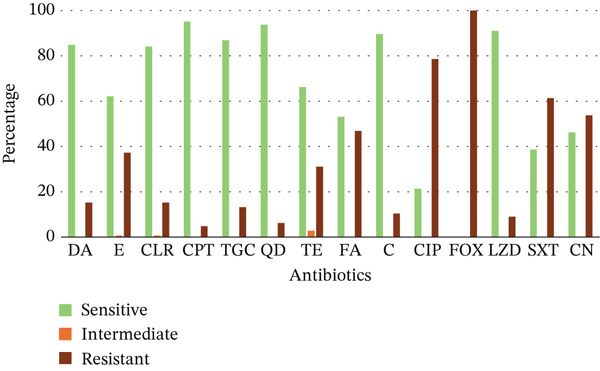
Antibiotic resistance profile of MRSA isolates used in the study.

MRSA was found to be highly resistant towards CIP (78.62%, *n* = 114), followed by SXT (61.38%, *n* = 89) and gentamicin (53.79%, *n* = 78). CPT possessed the highest susceptible rate of 95.17 (*n* = 138) (Figure 1).

### 3.3. SCCmec Typing

**Figure 2:**
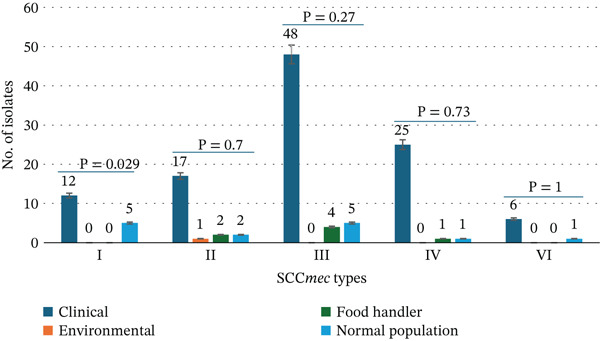
Distribution of SCC*mec* Types in hospital and community settings.

Out of 145 MRSA isolates, 15 (10.34%) were non‐typeable (NT) by the SCC*mec* typing protocol used. SCC*mec* Types I, II, III, IV, and VI were detected with no single Type V. The most prevalent SCC*mec* was Type III (39.31%, *n* = 57). Type IV was the least prevalent (4.83%, *n* = 7). Type I was significantly associated with the clinical isolates (*p* = 0.029) (Figure 2).

The comparison between antibiotic resistance in different SCC*mec* types revealed higher resistance in isolates harboring Type III with significant associated for ciprofloxacin phenotype (*p* < 0.001) (Table 3). Further, statistical analysis showed no significant difference in the distribution of SCC*mec* between CA‐ and HA (hospital‐acquired)‐MRSA. Table 4 shows binary logistic regression model to access the independent effect of SCC*mec* types on antibiotic resistance outcome.

**Table 3:** Association of SCC*mec* types and antibiotic resistance.

Antibiotics	Total resistant isolates	No. of resistant isolates in different SCC*mec* types (n)							
		I (*n* = 17)	II (*n* = 22)	III (*n* = 57)	IV (*n* = 27)	VI (*n* = 7)	NT (*n* = 15)	Chi‐square	*p* value
DA	20	2	4	8	6	0	2	2.63	0.622
E	49	4	10	22	12	1	5	4.19	0.38
CLR	21	3	5	8	4	1	1	0.97	0.914
CPT	7	1	0	2	4	0	0	6.77	0.149
TGC	17	1	1	8	5	2	2	4.41	0.353
QD	9	1	0	3	5	0	0	8.06	0.089
TE	43	7	7	15	11	3	2	0	1
FA	64	7	9	32	14	2	4	3.41	0.492
C	15	1	1	7	5	1	0	2.96	0.565
CIP	105	10	13	52	25	5	9	18.76	0.0008
LZD	13	0	2	8	3	0	0	3.76	0.44
SXT	83	8	11	41	17	6	6	6.98	0.137
CN	72	8	11	37	15	1	6	7.61	0.107

Abbreviations: C: chloramphenicol, CIP: ciprofloxacin, CLR: clarithromycin, CN: gentamycin, CPT: ceftaroline, DA: clindamycin, E: erythromycin, FA: fusidic acid, LZD: linezolid, QD: quinupristin‐dalfopristin, SXT: co‐trimoxazole, TE: tetracycline, TGC: tigecycline.

**Table 4:** Multivariate analysis for accessing independent effect of SCC*mec* types on antibiotic phenotype.

Antibiotic	SCC*mec* type	Adjusted OR	95% CI for OR	*p* value	Model *χ* ^2^ (df)	Model *p* value	Nagelkerke *R* ^2^
DA	I (Ref)	—	—	—	3.60 (4)	0.462	0.047
	II	2.16 × 10^8^	0.00–∞	0.999			
	III	3.59 × 10^8^	0.00–∞	0.999			
	IV	2.64 × 10^8^	0.000–∞	0.999			
	VI	4.62 × 10^8^	0.00–∞	0.999			
E	I (Ref)	—	—	—	5.319 (4)	0.256	0.054
	II	1.84	0.168–20.26	0.616			
	III	6	0.62–58.43	0.123			
	IV	3.77	0.46–33.47	0.233			
	VI	4.8	0.51–45.5	0.172			
CLR	I (Ref)	—	—	—	0.917 (4)	0.922	0.012
	II	1.286	0.110–15.003	0.841			
	III	0.765	0.170–18.321	0.634			
	IV	0.98	0.104–9.248	0.986			
	VI	1.043	0.098–11.144	0.972			
CPT	I (Ref)	—	—	—	6.932 (4)	0.14	0.152
	II	1.01 × 10^8^	0.00–∞	0.999			
	III	1	0.00–∞	1			
	IV	5.87 × 10^7^	0.00–∞	0.999			
	VI	2.81 × 10^8^	0.00–∞	0.999			
TGC	I (Ref)	—	—	—	4.609 (4)	0.33	0.065
	II	0.156	0.012–2.108	0.162			
	III	0.119	0.009–1.589	0.107			
	IV	0.408	0.067–2.474	0.33			
	VI	0.568	0.084–3.821	0.561			
QD	I (Ref)	—	—	—	8.440 (4)	0.077	0.159
	II	1 × 10^8^	0.00–∞	0.999			
	III	1	0.00–∞	1			
	IV	8.97 × 10^8^	0.00–∞	0.999			
	VI	3.67 × 10^8^	0.00–∞	0.999			
TE	I (Ref)	—	—	—	2.413 (4)	0.66	0.025
	II	0.933	0.157–5.543	0.939			
	III	0.923	0.165–5.162	0.927			
	IV	0.52	0.105–2.589	0.425			
	VI	0.917	0.170–4.930	0.919			
FA	I (Ref)	—	—	—	3.460 (4)	0.484	0.035
	II	1.75	0.261 – 11.737	0.564			
	III	1.731	0.273 – 10.974	0.56			
	IV	3.2	0.572 – 17.893	0.185			
	VI	2.692	0.443 – 16.373	0.282			
C	I (Ref)	—	—	—	3.161 (4)	0.531	0.047
	II	0.375	0.020–6.997	0.511			
	III	0.286	0.015–5.279	0.4			
	IV	0.84	0.088–8.049	0.88			
	VI	1.364	0.133–14.002	0.794			
CIP	I (Ref)	—	—	—	7.963 (4)	0.001	0.207
	II	0.571	0.085–3.833	0.564			
	III	0.578	0.091–3.663	0.56			
	IV	4.16	0.635–27.239	0.137			
	VI	5	0.564–44.343	0.148			
LZD	I (Ref)	—	—	—	6.042 (4)	0.196	0.095
	II	1.62 × 10^8^	0.00–∞	0.999			
	III	2.64 × 10^8^	0.00–∞	0.999			
	IV	2.02 × 10^8^	0.00–∞	0.999			
	VI	1	0.00–∞	1			
SXT	I (Ref)	—	—	—	7.103 (4)	0.131	0.073
	II	0.148	0.015–1.510	0.107			
	III	0.167	0.017–1.623	0.123			
	IV	0.427	0.048–3.833	0.447			
	VI	0.283	0.030–2.706	0.273			
CN	I (Ref)	—	—	—	7.993 (4)	0.092	0.08
	II	5.33	0.52–54.34	0.158			
	III	6	0.62–58.43	0.123			
	IV	11.1	1.25–98.76	0.031			
	VI	7.5	0.79–71.09	0.079			

Abbreviations: CI = confidence interval; df = degree of freedom; OR = odds ratio; Ref = reference group.

## 4. Discussion

The study’s strengths include the up‐to‐date prevalence of SCC*mec* types in the region, both in the community and hospital settings. Not only is the prevalence of SCC*mec* types I to IV re‐established, but the updated antibiotic resistance profiles of MRSA strains were also accessed about the prevalent SCC*mec* types. The present study found SCC*mec* type III as the most prevalent genotype in the study area, followed by type IV. These findings are supported by previous studies from Pakistan [19, 26]. Some recent studies from different regions of Pakistan have also reported type III as the most prevalent, with types I to V also [13, 27]. We did not find any type V isolates; however, type VI was the least prevalent (4.83%). This indicates that some local isolates frequently circulate in Pakistan [17]. In the last decade, previously conducted research has reported type I and II as the predominant SCC*mec* elements or the absence of type III [28, 29]. This evolution indicates the survival of successful isolates capable of developing resistance by uptake of resistance genes in the MGEs.

SCC*mec* typing revealed that 10.34% of isolates remained NT. Previous studies have also reported NT‐MRSA isolates for SCC*mec* typing [24, 30]. However, the quite high percentage of NT‐MRSA in the study isolates might indicate genomic variations in the circulating isolates. An in‐depth molecular surveillance study should be performed routinely to get an insight into the prevalent strains and avoid super bug development. Furthermore, to enhance the understanding of the local circulating strains and to fully explore the genomic background, the whole genome sequencing of the NT isolates is highly recommended in developing countries like Pakistan. In such locations, the pathogens are more prone to acquiring resistance mechanisms through horizontal gene transfer or mutations because of the mis and overuse of the antimicrobials.

Type III SCC*mec* type has been reported as the largest SCC*mec* element of all the types, thus harboring multiple antibiotic resistance genes [10, 31]. We also found the highest resistance in SCC*mec* type III, which was significantly associated with ciprofloxacin resistance (*p* < 0.001). A previous study has also reported a significant association of antimicrobial resistance with SCC*mec* types, including SCC*mec* type III, with ciprofloxacin and other antibiotics [32]. We found the highest prevalence of MRSA in pus from clinical isolates. At the same time, the nonclinical came from nares and hands, indicating these sources as predominant infectious sites and reservoirs of the pathogen, respectively. The logistic regression model also revealed that most of the SCC*mec* type harboring had a significant individual effect on antibiotic resistance (*p* > 0.05). However, comparing with the reference SCC*mec* I, the SCC*mec* III and VI had higher odds for DA and E, while type IV had higher odds for QD, FA, and CN. Type VI had higher odds for C and CIP. Despite statistical indications of some effect for Type VI in the univariate test (*p* = 0.008), the binary logistic model was not significant for the QD antibiotic. The linear regression model examining SCC*mec* and CIP resistance was statistically significant (*p* = 0.001), suggesting SCC*mec* types contribute meaningfully to CIP resistance prediction. Despite the significant model, individual SCC*mec* types were statistically significant, though types IV and VI showed elevated odds (OR = 4.16 and 5, respectively). This might be regarded as a trend but not a definitive association of CIP association with indicial SCC*mec* type.

Furthermore, all the CA isolates in the current study harbored the same SCC*mec* types as HA isolates, indicating horizontal gene transfer and evolution of the circulating strains. Another possible reason could be either the inappropriate use of antibiotics for minor infections or the irrational use of antibiotics in disinfectants post‐COVID‐19 pandemic era. Consequently, these potentially more pathogenic CA‐MRSA isolates harboring SCC*mec* elements could cause future human disaster if once able to bypass the normal flora barrier.

## 5. Conclusions

The multiresistant SCC*mec* type III was most predominant and significantly associated with antibiotic resistance. All the SCC*mec* types present in HA‐MRSA were also found in CA‐MRSA isolates, indicating the flow of the MGE across the community. Appropriate and immediate infection control policies and antibiotic stewardship programs should be implemented to prevent the spread and burden of antibiotic resistance in MRSA.

## Author Contributions

A.A.: conceptualization; writing – original draft; writing – review and editing; methodology; funding acquisition; investigation. S.J.: methodology; investigation; S.A.: review. S.R.: supervision; conceptualization; funding acquisition; validation; review.

## Funding

This study was supported by Higher Education Commission, Pakistan, 10.13039/501100004681.

## Ethics Statement

Ethical approval for this study was obtained from the Ethical Committee of the CitiLab & Research Centre Lahore, Pakistan (15A ‐ CLRC/30th).

## Conflicts of Interest

The authors declare no conflicts of interest.

## Data Availability

The data that support the findings of this study are available from the corresponding author upon reasonable request.
